# Nanostructured Clays Used as Carriers for Controlled Delivery of Antibacterial Compounds From Direct Restorative Dental Materials: A Scoping Review

**DOI:** 10.1002/cre2.70219

**Published:** 2025-09-08

**Authors:** Bárbara Faria Sa de Barbosa, José Leôncio Ferreira Neto, Francisca Jennifer Duarte de Oliveira, Juliana Sales Osterno Leitão, Moan Jéfter Fernandes Costa, Pedro Henrique Sette‐de‐Souza, Pierre Basílio Almeida Fechine, Victor Pinheiro Feitosa, Boniek Castillo Dutra Borges

**Affiliations:** ^1^ Department of Dentistry Universidade Federal do Rio Grande do Norte (UFRN) Natal Rio Grande do Norte Brazil; ^2^ Group of Chemistry of Advanced Materials (GQMat), Department of Analytical Chemistry and Physical Chemistry Universidade Federal do Ceará (UFC) Fortaleza Ceará Brazil; ^3^ College of Dentistry Universidade de Pernambuco (UPE) Arcoverde Pernambuco Brazil; ^4^ Department of Operative Dentistry, College of Dentistry University of Iowa Iowa City Iowa USA

**Keywords:** dental materials, dentistry, montmorillonite, nanoparticle drug delivery system

## Abstract

**Objective:**

Through a scoping review, this study meticulously mapped and characterized these nanostructured clays used to release antibacterial active compounds from direct restorative dental materials.

**Material and Methods:**

The systematic approach involved searches in the PubMed/MEDLINE, Lilacs, Web of Science, Scopus, ScienceDirect, and Embase databases. Two independent and calibrated researchers (kappa: 0.94) performed all systematic steps according to the PRISMA guideline and the Joanna Briggs Institute Scoping Review Methodology Group (JBI) protocol. The reviewers developed a data extraction table to gather key information.

**Results:**

A total of 782 articles were retrieved in the initial search using the specified strategies. After thoroughly reviewing the manuscripts, five were selected following the exclusion of duplicates and application of eligibility criteria. Montmorillonite and halloysite nanotubes emerged as the predominant nanostructured clay. Cetylpyridinium chloride was the most common active agent, notable for its antibacterial properties. Resin‐based composites were the most frequently studied direct restorative material for the prevention of recurrent caries.

**Conclusion:**

Although the number of primary studies published in the literature was limited, montmorillonite and halloysite nanotubes appear to be promising nanocarriers for antibacterial active compounds in direct restorative dental materials.

## 1 Introduction

In recent years, there has been a growing interest in developing innovative strategies to solve some limitations of direct restorative dental materials. Recurrent caries lesions are the main cause of restorations' failure, which leads to the need for restorations' replacement, causing loss of dental tissues during cavity preparation and public expenditure on public health services (Askar et al. 2017; Elmergawy et al. 2021). Factors such as the patient's caries risk profile, dietary habits, and oral hygiene practices are crucial determinants that influence susceptibility to caries development (Pitts et al. 2017). The complex interplay of intrinsic and extrinsic factors underscores the need for proactive approaches, such as incorporating antibacterial‐carrying capabilities into direct restorative dental materials, especially through a nanocarrier, to effectively mitigate the risk of caries around restorations and promote long‐term dental health (Khatoon et al. 2020).

Nanocarriers can allow the incorporation of functional compounds into dental materials, providing them with functional abilities (Costa et al. 2022). Controlled functional compound delivery systems have immense potential in dentistry, allowing the modulation of compounds' release kinetics to optimize therapeutic effects while minimizing adverse reactions (Villalba‐Rodríguez et al. 2021). Nanostructured clays, in particular, have garnered significant attention as potential carriers for the controlled delivery of functional compounds within dental materials, once they have good biocompatibility, high specific surface area, and interactions such as electrostatic and hydrogen bonds with the compound (Khatoon et al. 2020).

Montmorillonite (MMT), halloysite nanotubes (HNTs), and Laponite RD are some of the most common nanostructured clays, each with unique properties that make them suitable for a variety of applications (Gaharwar et al. 2019). MMT, a smectite clay mineral with a 2:1 structural arrangement, is commonly used in dental composites and adhesives (Ritto et al. 2022; Tsolianos et al. 2023). HNTs, with their hollow tubular structure, have been explored for gene and drug delivery, as well as for use in tissue engineering (Naumenko and Fakhrullin 2019; de Abreu Pereira et al. 2021, 2023). Laponite RD, a synthetic trioctahedral smectite, is particularly notable for its dual charge distribution, which makes it highly effective in enhancing the exfoliation and dispersion of nanoparticles within polymer matrices (Gaharwar et al. 2019).

Thus, nanostructured clays can offer the prospect of enhanced therapeutic outcomes and prolonged functional efficacy (Villalba‐Rodríguez et al. 2021). Once nanostructured clays can interact with different mineral and organic components, new opportunities arise for the design of functional direct restorative dental materials, such as resin‐based materials. Nanostructured clays can be designed to release therapeutic agents, such as antibacterial substances or drugs, in a controlled and sustained manner directly to the site of action (Villalba‐Rodríguez et al. 2021).

There are primary studies evaluating the ability of resin‐based materials to release drugs and functional substances, such as cetylpyridinium chloride and chlorhexidine, which were incorporated through nanostructured clays (Boaro et al. 2019; Matsuo et al. 2019; Barot et al. 2020; Yamamoto et al. 2022; Otsubo et al. 2023; Timbó et al. 2024). Despite the growing interest in drug delivery systems for dental applications using nanostructured clays, there is still no synthesis of evidence, such as a scoping review, that compiles the nanostructured clays used, proper concentrations, form of incorporation, and efficacy of the controlled release of functional compounds within direct restorative dental materials. The knowledge about the main nanostructured clays used and characterizing the process would help researchers plan further investigations.

The aim of this scoping review was to meticulously map and characterize the nanostructured clays used to carry functional compounds for controlled release in direct restorative dental materials, identifying key trends, gaps, and opportunities for further investigations.

## 2 Methods

### 2.1 Protocol and Registration

In this scoping review, the authors followed the Joanna Briggs Institute (JBI) protocol and reported according to the Preferred Reporting Items for Systematic Reviews and Meta‐Analyses extension for Scoping Reviews (PRISMA‐ScR) (Tricco et al. 2018).

The protocol was registered on the Open Science Framework platform under the DOI 10.17605/OSF.IO/9846M. With the research question “What are the nanostructured clays, and how are they used as carriers for controlled drug delivery in direct restorative dental materials?”, a search strategy was developed based on the Population, Concept, Context (PCC) strategy recommended for scoping reviews: Population – nanostructured clays; Concept – nanostructured clays used as carriers for controlled functional compounds delivery; and Context – direct restorative dental materials.

### 2.2 Eligibility Criteria

This study included primary studies using nanostructured clays as a carrier to control the release of functional compounds in direct restorative dental materials without publication date or language restrictions. Studies with absent data regarding the conjugation and incorporation processes were excluded.

### 2.3 Information Source

The search strategy was meticulously designed to include both published and unpublished studies. An initial, limited search of PubMed was conducted to identify relevant articles. The text words contained in the titles and abstracts of relevant articles and the indexing terms used to describe the articles were utilized to develop a comprehensive search strategy for the electronic databases. The database search was conducted without language or publication year restrictions until October 2024. Two previously trained authors (B.F.S.B. and J.L.F.N.) searched the following electronic databases: PubMed/MEDLINE, Lilacs, Web of Science, Scopus, ScienceDirect, and Embase, published until October 2024. The search strategy was adapted to meet the specific requirements of each database (Table 1).

**Table 1:** Specific search strategies for each database.

Database	Search Strategy	Results
PubMed	(“montmorillonite KSF”[Supplementary Concept] OR “Bentonite”[MeSH Terms] OR “Bentonite” OR “montmorillonite” OR “montmorillonites” OR “montmorillonitic” OR “Iaponite” OR “laponite based nano materials” OR “laponite clay” OR “laponite nanostructured clay” OR “Nanostructured clay”) AND (“Nanoparticle Drug Delivery System”[MeSH Terms] OR “Nanoparticle Drug Delivery System” OR “Nanotechnology” OR “Nanotechnology”[MeSH Terms] OR “Chemical Actions and Uses”[MeSH Terms] OR “Chemical Actions and Uses”) AND (“Dental Materials”[MeSH Terms] OR “Dental Materials” OR “Biomedical and Dental Materials”[MeSH Terms] OR “Biomedical and Dental Materials” OR “Dentistry”[MeSH Terms] OR “Dentistry” OR “Resin Composite” OR “Glass Ionomer” OR “Adhesive System” OR “Adhesive Systems” OR “Glass Ionomer Cements”[MeSH Terms] OR “Glass Ionomer Cements” OR “Dentin‐Bonding Agents”[MeSH Terms] OR “Dentin‐Bonding Agents” OR “Self‐Curing of Dental Resins”[MeSH Terms] OR “Self‐Curing of Dental Resins” OR “Light‐Curing of Dental Adhesives”[MeSH Terms] OR “Light‐Curing of Dental Adhesives”)	516
Emtree/Embase	(‘montmorillonite’/exp OR ‘bentonite’/exp OR ‘laponite’/exp OR ‘nanostructured clay’/exp) AND (‘nanocarrier’/exp OR ‘nanotechnology’/exp OR ‘drug activity’/exp) AND (‘dental material’/exp OR ‘dentistry’/exp OR ‘odontology’/exp OR ‘resin’/exp OR ‘glass ionomer’/exp OR ‘dentin bonding agent’/exp OR ‘dental self‐curing’/exp OR ‘adhesive agent’/exp)	49
BVS/LILACS	(“montmorillonite”) OR (mh:(“montmorillonite”)) OR (“bentonite”) OR (mh:(“bentonite”)) OR (“laponite”) OR (“nanostructured clay”) AND (mh:(“nanoparticle based drug delivery system”)) OR (“nanoparticle based drug delivery system”) OR (mh:(“nanotechnology”)) OR (“nanoparticle”) AND (“dental”) OR (mh:(“materials, dental”)) OR (“resin dental”) OR (mh:(“resin, dental”)) OR (“resin composite”) OR (mh:(“resins, composite”)) OR (“dentistry”) OR (mh:(“dentistry”)) OR (“odontology”) OR (“glass ionomer cements”) OR (mh:(“glass ionomer cements”)) OR (“dentin bonding agents”) OR (mh:(“dentin bonding agents”)) OR (“self‐cured dental bonding”) OR (mh:(“self‐curing of dental resins”)) OR (“self‐curing of dental resins”) OR (“light‐curing of dental adhesives”) OR (mh:(“light‐curing of dental adhesives”))	127
ScienceDirect	(“montmorillonite clay” OR nanostructured clay) AND (“nanoparticle based drug delivery system” OR nanocarrier OR nanotechnology) AND (dentistry OR “dental material”)	153
Scopus	(KEY (montmorillonite) OR KEY (bentonite) OR KEY (saponite) OR TITLE‐ABS‐KEY (montmorillonite) OR TITLE‐ABS‐KEY (organoclay) AND TITLE‐ABS‐KEY (Nanoparticle Drug Delivery System) OR KEY (Nanotechnology) OR KEY (Chemical Actions and Uses) AND KEY (Dental Materials) OR TITLE‐ABS‐KEY (Dentistry) OR TITLE‐ABS‐KEY (Resin Composite) OR TITLE‐ABS‐KEY (Glass Ionomer) OR TITLE‐ABS‐KEY (Adhesive Systems) OR TITLE‐ABS‐KEY (Dentin‐Bonding Agents) OR TITLE‐ABS‐KEY (Self‐Curing of Dental Resins) OR KEY (Light‐Curing of Dental Adhesives))	49
Web of Science	“Montmorillonite” OR “Bentonite” OR “Laponite” OR “Nanostructured clay” AND “Nanoparticle Drug Delivery System” OR “Nanoparticle” OR “Nanotechnology” OR “Chemical Actions and Uses” AND “Dental Materials” OR “Biomedical and Dental Materials” OR “Dentistry” OR “Resin Composite” OR “Glass Ionomer” OR “Glass Ionomer Cements” OR “Adhesive System” OR “Dentin‐Bonding Agents” OR “Self‐Curing of Dental Resins” OR “Light‐Curing of Dental Adhesives” OR “Odontology”	9

### 2.4 Search Strategy

The systematic searches (Table 1) were conducted based on Medical Subject Headings (MeSH) or the following specific text words: “montmorillonite”; “bentonite”; “iaponite”; “laponite based nano materials”; “laponite clay”; “laponite nanostructured clay”; “nanostructured clay”; “nanocarrier”; “nanoparticle drug delivery system”; “nanoparticle based drug delivery system”; “nanotechnology”; “chemical actions and uses”; “drug activity”; “dental materials”; “biomedical and dental materials”; “dentistry”; “resin”; “resin composite”; “glass ionomer”; “adhesive system”; “adhesive agent”; “glass ionomer cements”; “dentin‐bonding agents”; “self‐curing of dental resins”; “light‐curing of dental adhesives”.

The exhaustive search strategy across numerous databases aimed to gather a broad array of pertinent publications, minimizing the risk of overlooking crucial studies. Leveraging MeSH terms and precise keywords optimized the search process to retrieve studies concerning nanostructured clays used as carriers for controlled drug delivery and related subjects.

### 2.5 Selection of Evidence Sources

**Figure 1:**
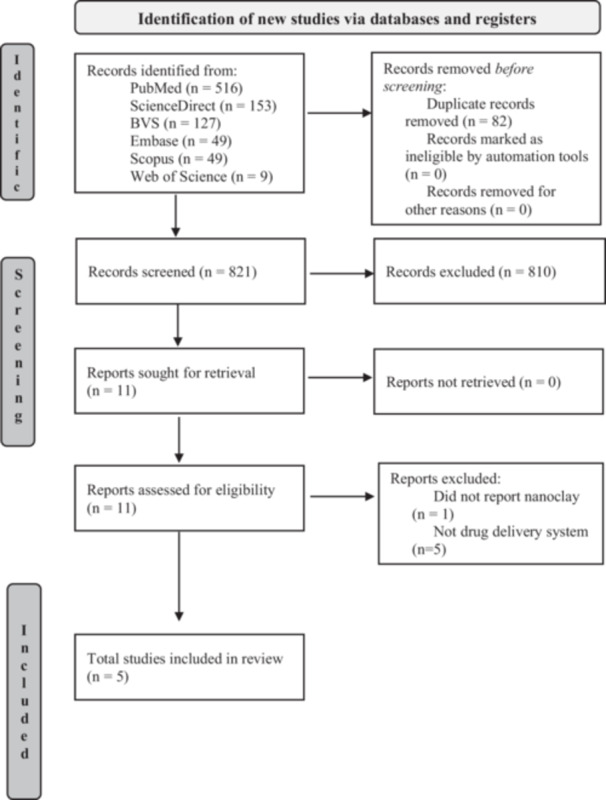
Flowchart with the entire process of identifying studies and databases.

For the organization and selection of studies, the Rayyan software (QCRI, Qatar Computing Research Institute; https://rayyan.qcri.org/) was employed to facilitate the screening and synthesis of articles. After removing duplicates, two authors (B.F.S.B. and J.L.F.N.), previously calibrated (*K* = 0.94), independently evaluated the titles and abstracts to exclude ineligible studies. Following a complete reading of the studies, articles that did not address the topics of the acronym, either in terms of functional compounds' release or description of the nanostructured clay used in the investigated material, were excluded, resulting in the removal of 6 articles. A flowchart was developed for the final selection of the studies to map and present the data found (Figure 1).

### 2.6 Data Charting Process

Following the final selection of articles, the reviewers (B.F.S.B. and J.L.F.N.) created a data extraction tool to gather information from each included source of evidence. They independently organized, discussed, and continually refined the data‐charting process. Any disagreements were resolved through discussion or involving a third reviewer (F.J.D.O).

Both reviewers independently extracted data using a standardized form in Microsoft Excel (Microsoft Corporation, Redmond, Washington, USA). The form captured key information relevant to the scoping review's questions. Any discrepancies were resolved through discussions between the two reviewers or, if necessary, by consulting a third reviewer for further clarification.

### 2.7 Data Extraction Process

Data were extracted from studies that met the inclusion criteria for analysis. The two reviewers developed a data extraction table to determine the key information. The data extracted from each article included: Authorship, date, and location of publication, title, study type, nanostructured clay used, concentration and weight of nanostructured clay, the functional compound used for release and its concentration, direct restorative dental material used in the study, method of functional compound incorporation, nanostructured clay surface modification, methods of agglutination, main results, and authors' conclusions.

The summary facilitated synthesizing the extracted data, allowing both reviewers and readers to understand the overall information and implications of the included studies.

### 2.8 Risk of Bias Assessment

The risk of bias assessment was performed using the Quality Assessment of in vitro studies (QUIN tool), which comprehends twelve domains: aims/objectives, sample calculation, sampling technique, details of groups, methodology explanation, operator details, randomization, method of outcome measurement, outcome assessor details, blinding, statistical analysis, and presentation of the results. Each domain was scored as “not specified” (score = 0), “inadequately specified” (score = 1), or “adequately specified” (score = 2). The RoB (%) was estimated using the following equation:

RoB(%)=(totalscore×100)/(two×numberofapplicablecriteria)

Studies were graded according to their obtained RoB as high risk (< 50%), medium risk (> 50% < 70%), or low risk (> 70%).

The primary studies included in the sample were analyzed independently by the two reviewers. Conflicts were solved by discussion with a third reviewer.

## 3 Results

### 3.1 Selection of Sources of Evidence

The database searches identified 903 studies. After removing duplicates, 821 studies remained (Figure 1). During the screening process, 810 reports were excluded based on their titles and abstracts. Subsequently, 11 potentially eligible studies were selected for further assessment, and their full texts were obtained in PDF format. After thoroughly evaluating these studies, 6 were excluded based on the eligibility criteria (Figure 1). Thus, five studies were eligible for inclusion in this scoping review synthesis.

### 3.2 Characteristics of Evidence Sources

The studies included in this scoping review were published in 2019 (*n* = 2), 2020 (*n* = 1), 2022 (*n* = 1), and 2023 (*n* = 1). The countries investigating this topic included Brazil (*n* = 1), India (*n* = 1), and Japan/Belgium (*n* = 3). Regarding the nature of the evidence, in vitro studies (*n* = 5) assessed experiments testing the development of new direct restorative dental materials. The investigated types of materials were resin composite (*n* = 3), adhesive system (*n* = 1), and dental cement (*n* = 1).

**Figure 2:**
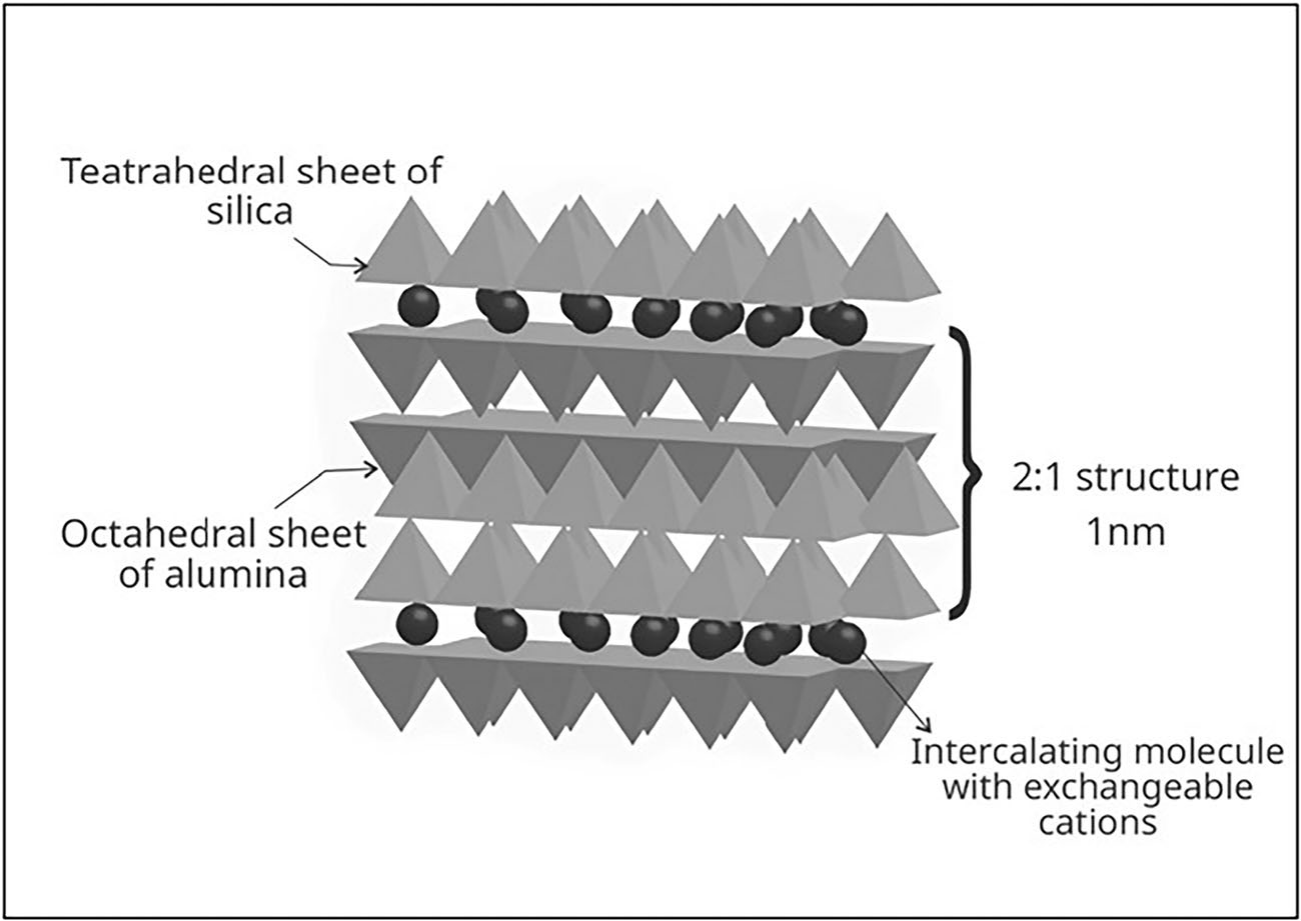
Schematic representation of the molecular structure of montmorillonite (MMT).

**Figure 3:**
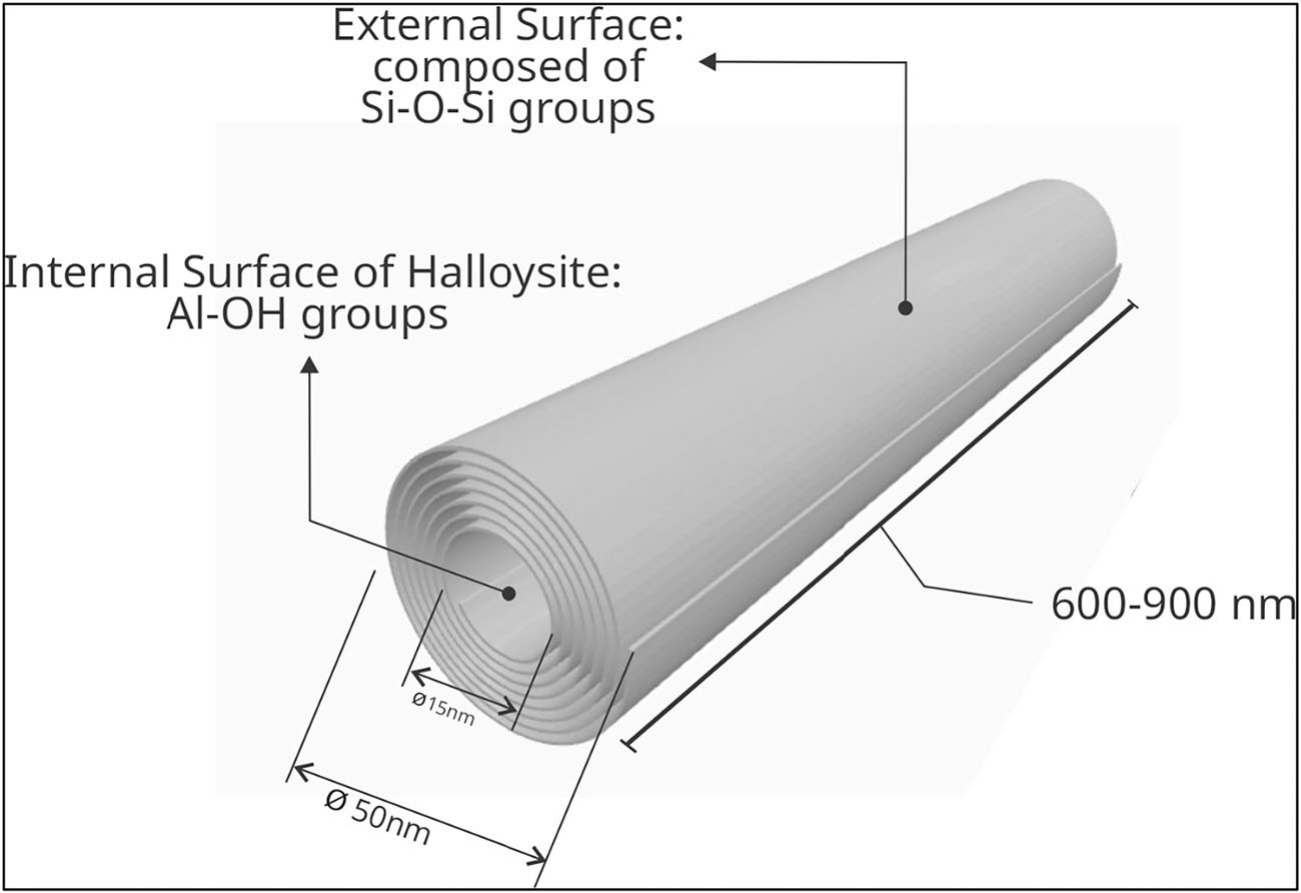
Schematic illustration of the molecular structure of halloysite nanotubes (HNTs).

The studies aimed to develop a functional compound delivery system within the tested materials. The researchers focused on nanostructured clays capable of entrapping antimicrobial agents and gradually releasing them in an aqueous media, aiming to prevent the onset of caries adjacent to the restorative material. Consequently, the nanostructured clays used in these studies were MMT (*n* = 4) (Figure 2) and HNTs (*n* = 1) (Figure 3).

Similarly, the studies retrieved in this review addressed three antimicrobial agents: farnesol (FA) (*n* = 1), chlorhexidine (CHX) (*n* = 1), and cetylpyridinium chloride (CPC) (*n* = 3). Given the variety of agents used, there was no consensus regarding the optimal concentration of these products to be released in an aqueous media.

Another way to improve the adhesion of the antimicrobial agent to the delivery vehicle is through the surface treatment of nanostructured clays. Various techniques have been employed to alter the structure of these nanomaterials to enhance their properties and efficacy as drug‐delivery vehicles. These modifications can be achieved through chemical processes, involving alterations in chemical composition and ion replacement (de Abreu Pereira et al. 2021). However, none of the articles included in this review reported surface treatments to improve the adhesion between antimicrobial agents and nanostructured clays.

### 3.3 Results of Individual Sources of Evidence

Barot et al. (2020) observed that incorporating the farnesol with halloysite nanotubes (Fa‐HNT system), with weight variations from 1 to 7 wt%, enhanced the mechanical properties of the dental resin composites. The addition of Fa‐HNT enhanced the mechanical properties of the resin compared to the control group. The mean flexural strength and compressive strength values were highest at a 7 wt% concentration, which was considered the optimal concentration. Scanning Electron Microscopy (SEM) images showed a uniform distribution of the nanostructured clay system and functional compound throughout the dental composite. The dental composite exhibited strong antimicrobial activity against *S. mutans*, with inhibition zones ranging from 6 to 13 mm. No cytotoxicity over 24, 48, and 72 h was observed, maintaining cell viability above 92%, which indicates its safety for use.

Matsuo et al. (2019) found that the release of CPC from 3 wt% adhesive formulations was superior to the 1 wt% formulation, noting the controlled release of the cetylpyridinium chloride with montmorillonite (CPC‐MMT system) over 10 days. Inhibition against *S. mutans* was observed for the 1 wt% and 3 wt% adhesive formulations. The 3 wt% formulation significantly reduced biofilm formation. Adequate cell viability was reported in cytotoxicity tests. With high magnification, using Field‐emission‐gun Scanning Electron Microscope (Feg‐SEM), the authors observed CPC‐MMT particles within the adhesive layers at the interfaces.

Boaro et al. (2019) demonstrated that CHX released from composites containing 2.5 wt% was higher than 5 wt% and 10 wt% over a 28‐day follow‐up. Micrographs displayed CHX incorporation into MMT and linear distribution in the specimen. All groups showed inhibition zones against *S. mutans, S. aureus*, and *P. gengivalis*, but only the specimens with 5% and 10% CHX exhibited antimicrobial activity against *P. gengivalis*. The CHX groups had greater inhibition compared to those with MMT alone. No statistical difference in biofilm formation was noted for groups of the same concentration containing either MMT or chlorhexidine with montmorillonite (CHX‐MMT system).

Otsubo et al. (2023) reported a gradually decreasing release of CPC from self‐curing dental resin specimens. The group with the highest sustained release of CPC was CPC‐MMT in Days 1–5, after which all groups exhibited similar release profiles. Tests showed that the CPC‐MMT group was able to reabsorb CPC and release it in a sustained manner, with a peak on the seventh day. Samples containing CPC‐MMT displayed greater turbidity, indicating antimicrobial efficacy. The mechanical strength decreased with the addition of MMT (CPC‐MMT group), reducing flexural strength by 36% compared to the control. The CPC‐MMT group also showed pigmentation compared to the control, with noticeable color changes in all evaluations.

Yamamoto et al. (2022) using a dual‐cure composite cement, observed that there were no significant changes in mechanical properties when adding a 2 wt% CPC‐MMT system. CPC release was observed over 7 days, being highest on the first day, but continuing at a decreased concentration thereafter. Regarding biofilm formation, it was noted that no biofilm formed at all concentrations of CPC‐MMT.

### 3.4 Synthesis of Results

Overall, nanostructured clays have proven effective as a carrier for controlled delivery of functional compounds in direct restorative dental materials. All studies observed good bacterial inhibition and biofilm reduction levels despite the different concentrations of functional compounds and nanostructured clays. A common concentration range of 3% to 10% of active agent incorporated into the nanoclay was observed across all studies, with concentrations between 5% and 7.5% consistently yielding the most favorable outcomes in all evaluated tests. All formulations in the selected studies demonstrated controlled release of the functional compounds and maintained their important physical and chemical properties for direct restorative dental materials. The controlled release was observed over several days and began reducing its effect after a maximum of 28 days. The data extraction of primary studies included in this scoping review can be found in Tables 2 and 3.

**Table 2:** General data extraction from primary studies.

References	Nanostructured clay	Carried compound	Nanostructured clay surface modification	Method of agglutination	Final preparation concentrations	Dental material	Tested groups	Methods of characterization	Main results
Barot et al. (2020)	HNT	FA	Not reported	Solubilization ‐ ethanol solution	0,02 g/mL (total filler load maintained at 50 wt%).	Resin composites ‐ 50 wt % of dimethacrylate‐based resin matrix (Bis‐GMA/TEGDMA ‐ 50/50 wt/wt. Respectively, with 0.5 wt % CQ and 0.5 wt% EDMAB as a photoinitiator system).	Control group and seven groups containing different mass fractions of Fa‐HNT (1, 3, 7, 10, 13, 17 and 20 wt%).	Identification of the nanostructured clay with the functional compound through surface morphology (SEM); Internal structure visualization (TEM); Chemical bond vibrations (FT‐IR); The crystalline structure of the system (XRD); Flexural Strength (UTM); Flexural Modulus (UTM); Compressive Strength (UTM); Degree of conversion (FT‐IR); Curing depth (DC); Antimicrobial activity (DDA); Cytotoxicity (MTT).	SEM images revealed that most specimens are polydisperse cylindrical tubes, measuring between 100 and 200 nm in length; Specimens observed under TEM reveal a hollow, open‐ended tubular structure of nanotubes; After loading FA, the entrapment efficiency percentage in the internal lumen was determined to be 74%; The diffraction analysis indicates that loading Farnesol into HNTs does not alter the crystalline structures of the HNTs; The incorporation of HNT‐based fillers in dental composites significantly improved both flexural and compressive strengths, with optimal results observed at a 7 wt% concentration of FA‐HNT filler. However, increasing the filler content beyond this level (13–20 wt%) led to a decline in mechanical properties; As the concentration of FA‐HNT increased from 1 to 10 wt%, the DC values decreased, but this trend intensified at concentrations of 13–20 wt%; The sequential addition of FA‐HNT filler (1–20 wt%) in dental composites led to a gradual decrease in curing depth, likely due to the higher refractive index of HNTs; Antibacterial activity against *S. mutans* increased with FA‐HNT concentration, peaking at 20 wt%; Fa‐HNT composites also showed minimal cytotoxicity on NIH‐3T3 cells.
Boaro et al. (2019)	MMT	CHX	Not reported	Solubilization ‐ aqueous solution	1 mg/100 mL	Resin composite ‐ organic phase (Bis‐GMA/TEGDMA ‐ 50/50 wt/wt.) Photoinitiator system (DMAEMA 1%/camphorquinone 0.8% (w/w)).	Three experimental resin composites containing MMT particles at 2.5, 5% and 10% (w/w), and three experimental resin composites with CHX/MMT at 2.5, 5% and 10% (w/w).	Decomposition profile of MMT particles (TGA); The type of interaction between MMT particles and CHX (XRD); Surface morphology and the particle size distribution (SEM); Degree of conversion (FT‐IR); Flexural strength (UTM); Internal structure visualization (TEM); Evaluation of the release of CHX (UV spectrophotometry); Antibacterial activity (AM; BF).	Resin composite with improved thermal stability, but a mass loss around 7% higher; The XRD suggests the incorporation of CHX between the MMT layers. The particles presented the same pattern in both cases (with or without CHX); The degree of conversion ranged from 71% to 74%; The flexural modulus ranged from 5.7 to 8.1 GPa; The flexural strength ranged from 61.4to 74.7 MPa. The resin composite containing 2.5 wt% MMT/CHX presented higher chlorhexidine release than 5 and 10 wt%. The specimens released CLX during the 28 days. The resin composite showed potential as a dental material with local antibacterial activity against various oral bacteria, including *S. mutans, P. gingivalis*, and *S. aureus*, while also reducing biofilm formation.
Matsuo et al. (2019)	MMT	CPC	Not reported	Solubilization ‐ aqueous solution	1 g/100 mL	Adhesive ‐ The one‐step self‐etch adhesive was modified by adding 1 wt% and 3 wt% CPC‐MMT, 3 wt% MMT (without CPC) and 1 wt% and 3 wt% CPC (without MMT)	Five experimental groups: 1 wt% and 3 wt% CPC‐MMT, 3 wt% MMT (without CPC) and 1 wt% and 3 wt% CPC (without MMT)	The crystalline structure of the system (XRD); CPC release (UV‐vis); Biofilm formation (Feg‐SEM); Cytotoxicity (MTT); Degree of conversion (FT‐IR); Micro‐tensile dentin bond strength (μTBS); Adhesive‐dentin interfacial visualization (TEM); Rechargeability CPC (XRD).	XRD analysis confirmed CPC incorporation into MMT and detailed its release mechanism and the result indicating high crystallinity; Feg‐SEM revealed at relatively low magnification irregular globular particles of MMT varying in size; The 3 wt% CPC/MMT adhesive formulation released more CPC than 1 wt%, and occurred for 10 days. 3 wt% CPC reduced biofilm formation; Cell viability decreased for all experimental conditions compared to the control (no disk); Only the 3 wt% CPC adhesive formulation (without MMT) showed a significantly lower degree of conversion; All tested adhesive formulations showed no significant difference in immediate dentin μTBS; TEM analysis showed tight adhesive‐dentin interfaces for all formulations; Significant *S. mutans* growth was observed before recharge, but no growth occurred afterward.
Otsubo et al. (2023)	MMT	CPC	Not reported	Solubilization – aqueous solution	10 wt%	Self‐curing resin ‐ copolymer of methacrylic ester; methyl methacrylate; tertiary amine; pigment.	Three experimental groups: Resin‐MMT; Resin‐NPS; Resin‐CPC (control)	Antimicrobial agent sustained release capacity (HPLC); Reuptake capacity (LC); Antimicrobial efficacy (S); Mechanical Strength ‐ three‐point bending test (UTM); Color change (CR‐20).	The sustained release of CPC decreased gradually across all specimens, with Resin‐MMT showing higher release than resin‐NPS from days 1–5, but both became nearly equal after day 6; The amount of CPC sustained release decreased gradually in the same manner as before reuptake; Resin‐MMT and Resin‐NPS groups showed sustained CPC release and effective reuptake. They released CPC against Streptococcus mutans for 14 days and post‐reuptake; MMT reduced mechanical strength, with resin‐Mont showing a 36% decrease in flexural strength, compared to control; The color difference of all specimens on day 0, day 7, and day 7 after reuptake exceeded the just‐noticeable difference compared to the control.
Yamamoto et al. (2022)	MMT	CPC	Not reported	Solubilization ‐ aqueous solution	1 g/100 mL 10.s5 g/250 mL	Resin cement ‐ The base paste was modified by adding either CPC‐MMT30 or CPC‐MMT7 (Different particle sizes CPC‐MMT with a median diameter of 30 μm and 7 μm) at concentrations of 2, 3, 4, 5 and 7.5 wt% in respect to the cement's total weight.	Ten experimental groups: CPC‐MMT30 or CPC‐MMT7 at concentrations of 2, 3, 4, 5 and 7.5 wt%	Flexural strength, elastic modulus and fracture toughness (UTM); Micro‐tensile bond to dentin (μTBS); CPC release (UV‐vis); Biofilm formation (SEM).	The 7.5 wt% CPC‐MMT30 had significantly lower mean flexural strength than the control. The elastic modulus varied between 3.8 to 4.9 GPa with no significant differences among cement formulations. However, fracture toughness notably decreased with the addition of CPC‐MMT30. In contrast, CPC‐MMT7 formulations showed no significant reductions in mechanical Properties; Bond strength decreased as CPC‐MMT30 concentration increased, with the 7.5 wt% CPC‐MMT30 formulation showing the lowest μTBS; Both the experimental CPC‐MMT30 and CPC‐MMT7 resin cement formulations presented a similar tendency in CPC‐release behavior; Adding 5‐7.5 wt% CPC‐MMT7 preserved properties and provided ongoing anti‐biofilm activity, with no biofilm formation observed on 5 wt% and 7.5 wt% CPC‐MMT7 disks after 30 days.

Abbreviations: MMT, montmorillonite; HNT, halloysite nanotubes; NPS, nanoporous silica; CPC, cetylpyridinium chloride; CHX, chlorhexidine; FA, farnesol; XDR, X‐ray diffractometer; SEM, scanning electron microscopy; FT‐IR, infrared spectrophotometer with Fourier Transform; TEM, transmission electron microscopy; UTM, Universal Testing Machine; DC, digital calliper; DDA, disk diffusion assay; AM, agardilution method; BF, biofilm Formation; TGA, thermogravimetry analysis; MTT, MTT ((3‐[4,5‐dimethylthiazol‐2‐yl]‐2,5 diphenyl tetrazolium bromide) assay/cytotoxicity test; μTBS, micro‐tensile testing device; UV–vis, UV–vis Spectroscopy; HPLC, high performance liquid chromatography; LC, liquid chromatography; S, spectrophotometer; CR‐20, portable colorimeter.

**Table 3:** Quantitative data regarding mechanical and antimicrobial tests extracted from primary studies according to the groups evaluated.

References	Flexural strength (MPa) (Mean ± SD)	Elastic modulus (GPa) (Mean ± SD)	Flexural modulus (GPa) (Mean ± SD)	Microtensile bond strength (MPa) (Mean ± SD)	Compressive strength (MPa) (Mean ± SD)	Fracture toughness (MPa·m¹/²) (Mean ± SD)	Cytotoxicity (% viability) (Mean ± SD)	Antimicrobial efficacy	Degree of conversion (DC) (Mean ± SD)
Barot et al. (2020)	Control (104.2 ± 0.00) (a) Fa‐HNT 1% (RF) (b) Fa‐HNT 3% (RF) (b) Fa‐HNT 7% (115.9 ± 2.07) (c) Fa‐HNT 10% (RF) (d) Fa‐HNT 13% (RF) (d) Fa‐HNT 17% (RF) (d) Fa‐HNT 20% (RF) (d) Results: c > b > a > d	Not evaluated	Control (6.50 ± 0.00) Fa‐HNT 1% (RF) Fa‐HNT 3% (RF) Fa‐HNT 7% (RF) Fa‐HNT 10% (RF) Fa‐HNT 13% (RF) Fa‐HNT 17% (RF) Fa‐HNT 20% (RF) Results: no statistically significant differences among the groups	Not evaluated	Control (280.5 ± 0.00) (b) Fa‐HNT 1% (RF) (a) Fa‐HNT 3% (RF) (a) Fa‐HNT 7% (310. 60 ± 2.40) (a) Fa‐HNT 10% (RF) (c) Fa‐HNT 13% (RF) (c) Fa‐HNT 17% (RF) (c) Fa‐HNT 20% (RF) (c) Results: a > b > c	Not evaluated	Control (100) Fa‐HNT 1% (RF) Fa‐HNT 3% (RF) Fa‐HNT 7% 24 h: (92,3); 48 h: (97,8); 72 h: (100) Fa‐HNT 10% (RF) Fa‐HNT 13% (RF) Fa‐HNT 17% (RF) Fa‐HNT 20% (RF) Results: no statistically significant differences among the groups	Not evaluated	Control (RF) Fa‐HNT 1% (RF) Fa‐HNT 3% (RF)) Fa‐HNT 7% (RF) Fa‐HNT 10% (RF) Fa‐HNT 13% (RF) Fa‐HNT 17% (RF) Fa‐HNT 20% (RF)
Boaro et al. (2019)	Resin matrix (control) (66.4 ± 15.5) 2.5% MMT (61.4 ± 15.0) 2.5% MMT/CHX (71.0 ± 17.6) 5% MMT (61.9 ± 17.8) 5% MMT/CHX (62.1 ± 8.8) 10% MMT (74.7 ± 17.4) 10% MMT/CHX (69.9 ± 10.0)	Resin matrix (control) (8.1 ± 1.76) 2.5% MMT (7.5 ± 1.55) 2.5% MMT/CHX (7.1 ± 1.62) 5.0% MMT (7.6 ± 2.04) 5.0% MMT/CHX (6.4 ± 1.23) 10.0% MMT (5.7 ± 1.28) 10.0% MMT/CHX (6.9 ± 2.00)	Not evaluated	Not evaluated	Not evaluated	Not evaluated	Not evaluated	Not evaluated	(Measured after 10 min) Resin matrix (74 ± 7) 2.5% MMT (71 ± 5) 2.5% MMT/CHX (74 ± 8) 5% MMT (73 ± 7) 5% MMT/CHX (74 ± 3) 10% MMT (71 ± 5) 10% MMT/CHX (72 ± 6)
Matsuo et al. (2019)	Not evaluated	Not evaluated	Not evaluated	1% CPC_Mont (RF) 3% CPC_Mont (RF) 1% CPC (RF) 3% CPC (RF) Only 3 wt% CPC had significantly reduced immediate and aged bond strength	Not evaluated	Not evaluated	Cytotoxicity (MTT assay) Mont (RF) C‐S3B (RF) 1% CPC_Mont (RF) 3% CPC_Mont (RF) 1% CPC (RF) 3% CPC (RF) 3 wt% CPC significantly more cytotoxic than other groups	Not evaluated	Mont (RF) C‐S3B (RF) 1% CPC_Mont (RF) 3% CPC_Mont (RF) 1% CPC (RF) 3% CPC (RF) Only 3 wt% CPC had lower polymerization conversion than other adhesives
Otsubo et al. (2023)	Resin‐cont (85.8 ± RF) Resin‐NPS (64.0 ± RF) Resin‐Mont (54.9 ± RF) Resin‐CPC (59.2 ± RF) There is a statistically significant difference (*p* < 0.05) between the control group (resin‐cont) and all other groups	Not evaluated	Not evaluated	Not evaluated	Not evaluated	Not evaluated	Not evaluated	Control (RF) Resin‐Mont (RF) Resin‐NPS (RF) Resin‐CPC (RF) No statistical difference explicitly reported	Not evaluated
Yamamoto et al. (2022)	0 wt% Control CPC‐Mont30 (RF) 2 wt% ‐ CPC‐Mont30 (RF) 3 wt% ‐ CPC‐Mont30 (RF) 4 wt% ‐ CPC‐Mont30 (RF) 5 wt% ‐ CPC‐Mont30 (RF) 7.5 wt% ‐ CPC‐Mont30 (45.6 ± 6.9) Lower statistically significant difference than control, 2%, 3% and 4% CPC‐Mont30 CPC‐Mont7‐Controle: (RF) 2 wt% ‐ CPC‐Mont7 (RF) 3 wt% ‐ CPC‐Mont7 (RF) 4 wt%‐ CPC‐Mont7 (RF) 5 wt% ‐ CPC‐Mont7 (RF) 7.5 wt% ‐ CPC‐Mont7 (RF)	0 wt% Control CPC‐Mont30 (RF) 2 wt% ‐ CPC‐Mont30 (RF) 3 wt% ‐ CPC‐Mont30 (RF) 4 wt% ‐ CPC‐Mont30 (RF) 5 wt% ‐ CPC‐Mont30 (RF) 7.5 wt% ‐ CPC‐Mont30 (RF) CPC‐Mont7‐Controle: (RF) 2 wt% ‐ CPC‐Mont7 (RF) 3 wt% ‐ CPC‐Mont7 (RF) 4 wt%‐ CPC‐Mont7 (RF) 5 wt% ‐ CPC‐Mont7 (RF) 7.5 wt% ‐ CPC‐Mont7 (RF) No statistically significant differences among groups	Not evaluated	0 wt% Control CPC‐Mont30 (RF) 2 wt% ‐ CPC‐Mont30 (RF) 3 wt% ‐ CPC‐Mont30 (RF) 4 wt% ‐ CPC‐Mont30 (RF) 5 wt% ‐ CPC‐Mont30 (RF) 7.5 wt% ‐ CPC‐Mont30 (6.4 ± 3.2) CPC‐Mont7‐Controle: (RF) 2 wt% ‐ CPC‐Mont7 (RF) 3 wt% ‐ CPC‐Mont7 (RF) 4 wt%‐ CPC‐Mont7 (RF) 5 wt% ‐ CPC‐Mont7 (RF) 7.5 wt% ‐ CPC‐Mont7 (RF) CPC‐Mont30 – 2 wt%, 7.5 wt% and CPC‐Mont30 ‐ 7,5 wt% showed significantly lower fracture toughness compared to the control	Not evaluated	Fracture toughness (MPa·m¹/²) 0 wt% Control CPC‐Mont30 (RF) 2 wt% ‐ CPC‐Mont30 (RF) 3 wt% ‐ CPC‐Mont30 (RF) 4 wt% ‐ CPC‐Mont30 (RF) 5 wt% ‐ CPC‐Mont30 (RF) 7.5 wt% ‐ CPC‐Mont30 (RF) CPC‐Mont7‐Controle: (RF) 2 wt% ‐ CPC‐Mont7 (RF) 3 wt% ‐ CPC‐Mont7 (RF) 4 wt%‐ CPC‐Mont7 (RF) 5 wt% ‐ CPC‐Mont7 (RF) 7.5 wt% ‐ CPC‐Mont7 (RF) CPC‐Mont30 – 2 wt% and 7.5 wt% showed significantly lower fracture toughness compared to the control	Not evaluated	Not evaluated	Not evaluated

Abbreviations: MMT, montmorillonite; HNT, halloysite nanotubes; NPS, nanoporous silica; CPC, cetylpyridinium chloride; CHX, chlorhexidine; FA, farnesol; SD, standard deviation; Fa‐HNT, farnesol incorporated halloysite nanotubes; RF, the presentation in the figure did not allow for the exact numerical extraction of the data (mean ± SD); MMT, montmorillonite; MMT/CHX, chlorhexidine‐incorporated montmorillonite; CPC_Mont, cetylpyridinium chloride incorporated montmorillonite; CPC, cetylpyridinimmum chloride; Resin‐Mont, autopolymerizing resin containing montmorillonite (Mont) loaded with cetylpyridinium chloride (CPC); Resin‐NPS, autopolymerizing resin containing nanoporous silica (NPS) also loaded with CPC; Resin‐CPC, autopolymerizing resin with CPC directly added, without using a carrier such as Mont or NPS; CPC‐Mont30, cetylpyridinium chloride incorporated montmorillonite with an average diameter of 30 µm; CPC‐Mont7, cetylpyridinium chloride incorporated montmorillonite with an average diameter of 7 µm.

### 3.5 Risk of Bias Assessment

In the risk of bias analysis, all the articles were classified as medium risk. The most penalized domains were C2, C6 and C7. In domain C2, all studies were classified with zero because the authors did not mention a sample size calculation. Domains C6 and C7 received zero ratings due to a lack of operator information and sample randomization (Table 4).

**Table 4:** Risk of bias assessment of included studies.

Study	C1	C2	C3	C4	C5	C6	C7	C8	C9	C10	C11	C12	FS	%RoB	RoB
Barot et al. (2020)	2	0	2	2	2	0	0	2	1	NA	1	2	14	63%	Medium risk
Boaro et al. (2019)	2	0	2	2	2	0	0	2	1	NA	2	2	15	68%	Medium risk
Matsuo et al. (2019)	2	0	2	2	2	0	0	2	1	NA	2	2	15	68%	Medium risk
Otsubo et al. (2023)	2	0	1	2	2	0	0	2	1	NA	0	2	12	54%	Medium risk
Yamamoto et al. (2022)	2	0	2	2	2	0	0	2	1	NA	2	2	15	68%	Medium risk

*Note:* QUIN Tool's criteria: C1, clearly stated aims/objectives; C2, detailed explanation of sample size calculation; C3, detailed explanation of sampling technique; C4, details of comparison group; C5, detailed explanation of methodology; C6, operator details; C7, randomization; C8, method of measurement of outcome; C9, outcome assessor details; C10, blinding; C11, statistical analysis; C12, presentation of results; N/A, not applicable; FS, final score; %RoB, percentage of risk of bias; RoB, risk of bias. %RoB < 50% high risk, 50% < %RoB < 70% medium risk, %RoB > 70% low risk.

## 4 Discussion

This scoping review explored and characterized nanostructured clays used as carriers for the controlled delivery of functional compounds in direct restorative dental materials. Thus, the type of nanostructured clay chosen, the functional compound used and its function, and the compound‐delivery system produced were extensively mapped and analyzed.

Developing functional restorative materials has gained efforts in the field of dental materials. Caries lesions adjacent to direct restorative dental materials, such as resin composites, have been considered the main cause of restoration failure (Askar et al. 2021). Once caries is a biofilm‐dependent disease, researchers in the dental materials field are motivated to develop materials with antibacterial properties, which might reduce the incidence of recurrent caries and restoration replacement (Barot et al. 2020).

A significant adjunct in the development of materials with antibacterial properties is nanocarriers, which have shown great potential for use in various fields, with the strategy of safely delivering local functional compounds, compatible with the requirements of each presentation form (Dong et al. 2021). Nanostructured clays stand out as nanomaterials with a layered structure, which allows for the intercalation and adsorption of molecules within their framework (Elmergawy et al. 2021; Dong et al. 2021). Additionally, most nanostructured clays possess hydrophilic properties and a high cation exchange capacity, facilitating interaction with different functional compounds and their controlled release (Park et al. 2016). The difference in the morphology of nanostructured clays influences functional compound loading, with the reduction in clay dimensions to the nanoscale improving performance, availability, and controlled release (Dong et al. 2021). These nanostructured clays are ultra‐thin polar materials on the nanometer scale (Villalba‐Rodríguez et al. 2021).

To improve the binding of functional compounds with nanostructured clays, surface modification might be a promising strategy. These modifications can enhance substance attachment, increase stability, and reduce nonspecific interactions (Li et al. 2019). Differences in the internal and external surfaces of nanostructured clays may create distinct compositions, fostering stronger interactions between the nanostructured clays and functional compounds (Riela et al. 2014). Techniques such as chemical processes, thermal treatments, or mechanical alterations that modify particle size and surface properties can be applied for this purpose (de Abreu Pereira et al. 2021). However, none of the studies reviewed in this paper employed surface treatments to boost adhesion or stabilize these interactions. The functional compounds tested in the primary studies likely had great affinity to the nanostructured clays tested.

This scoping review identified that MMT had been the most commonly used nanostructured clay for delivering functional compounds in direct restorative dental materials. MMT (Figure 2) is a type of aluminosilicate nanostructured clay composed of crystalline layers formed by silicon oxide and aluminum hydroxide with hydrophilic properties and a high cation exchange capacity (Elmergawy et al. 2021; Dong et al. 2021). This cationic capacity is higher compared to other silicates, and its layers provide ample space for molecule binding, an important characteristic of compound adhesion (Dong et al. 2021). Due to the affinity of MMT for polar molecules (Park et al. 2016), such as chlorhexidine digluconate and CPC, its adsorption capacity is increased, which contributes to the retention and controlled release of functional compounds with polar characteristics. Thus, MMT exhibits hydrophilic characteristics, showing swelling in the presence of water, which contributes to the formation of a gelatinous material when combined with functional compounds (Ritto et al. 2022; Dong et al. 2021; Park et al. 2016).

Another nanostructured clay identified as a carrier for functional substances in the present study was HNTs (Figure 3). They are tubular‐shaped, two‐layered aluminosilicate structures, presenting a porous appearance (Barot et al. 2020; Fizir et al. 2018). HNTs have aluminol on the inner side and siloxane on the outer side, which confer positive and negative charges, respectively (Timbó et al. 2024), allowing conjugation with polar and apolar compounds. The stability of the halloysite surface is due to the presence of anionic organic substances. The surface of HNT has selectivity that allows for various surface treatments to enhance properties such as porosity and surface contact area, which facilitate the incorporation of active agents for controlled release (de Abreu Pereira et al. 2023). Both MMT and HNTs can be employed in the loading and delivery of functional compounds, as they are considered significantly safe, with low cytotoxic potential, and biocompatible (Ritto et al. 2022; Barot et al. 2020). Due to these characteristics, these two nanostructured clays were the primary choices as carrier functional compounds in direct restorative dental materials. Although both nanostructured clays play an excellent role as functional compound carriers, MMT loaded with chlorhexidine (Boaro et al. 2019) was able to release the active ingredient for 28 days, which was the longest duration observed in the experiment. This may be a favorable characteristic for choosing the MMT, besides its chemical compatibility with CLX. On the other hand, HNTs present a moderate increase in the tube diameter after loading with farnesol (Barot et al. 2020), which led to a decrease in mechanical properties. Thus, the agglomeration of HNTs may hinder the interaction between the nanostructured clay and the polymeric matrix.

Among the functional compounds used in the analyzed studies, cetylpyridinium chloride (CPC) was the most prevalent (60% of studies). CPC is a monocationic compound of quaternary ammonium (QAC), which is also a cationic surfactant similar to chlorhexidine (CHX) with broad‐spectrum antimicrobial activity against bacteria and fungi (Mao et al. 2020; Urakawa et al. 2020; Brezhnev et al. 2023). CPC is an antimicrobial ingredient commonly used in over‐the‐counter products, aiming to reduce biofilm accumulation and gingival inflammation (Mao et al. 2020; Urakawa et al. 2020). Another factor justifying the use of CPC is its good solubility in water, which facilitates manipulation with nanostructured clays, which, as previously shown, are materials with excellent adsorption properties to polar molecules (Mao et al. 2020). Once CPC is less affected by dilution and saliva (Urakawa et al. 2020), it is indicated for oral dosing with sufficient antimicrobial effect. The data showed in the primary studies included in the present scoping review evidenced that CPC has an antibacterial effect against caries‐related microorganisms (Matsuo et al. 2019; Yamamoto et al. 2022; Otsubo et al. 2023). Previous investigations (Mao et al. 2020; Smith et al. 2013) in oral mouthwashes further support CPC's effectiveness, showing that even at low concentrations, below 1%, CPC is effective against *S. mutans* and methicillin‐resistant *Staphylococcus aureus* (MRSA), common in the oral microbiota associated with dental caries.

Data analysis revealed that two other important antimicrobial agents, farnesol and chlorhexidine, were identified in the primary studies included in this scoping review, each representing 20% of the total studies. Chlorhexidine is an antimicrobial agent widely used in various medical areas, such as dermatology and surgery, as well as in dentistry, due to its bactericidal action, aiding in biofilm reduction and gingival inflammation (Liu et al. 2023). Additionally, it is considered the gold standard of oral antiseptics due to its bactericidal and bacteriostatic activities (Arathi et al. 2019). CLX is used in saline forms such as acetate, digluconate, and dihydrochloride to allow miscibility in water (Chronopoulou et al. 2016), and its conjugation to MMT. Conversely, farnesol is a naturally occurring organic compound certified as GRAS (Generally Regarded as Safe), used as a flavoring agent and food additive, which has three carbon–carbon double bonds and exists in four isomers. Farnesol exhibits lipophilic properties and antifungal and antibacterial effects (Barot et al. 2020; Hornby et al. 2001; Horev et al. 2015).

Nanocarriers have proven to be an excellent approach to allow localized and controlled delivery of functional compounds. After mapping the literature, MMT and HNTs emerged as a prominent choice as a nanocarrier by effectively entrapping and gradually releasing the functional compounds, owing to their unique properties such as layered structure and cation exchange capacity. Other non‐nanoclay delivery systems, such as polymeric nanoparticles loaded with chlorhexidine, have emerged as a viable strategy for the sustained release of antimicrobial agents for 28 days. This performance results from the combination of high encapsulation efficiency, small particle size with uniform morphology, and effective in vitro controlled release behavior (Priyadarshini et al. 2017). Thus, further synthesis studies can focus on comparing the efficacy of nanoclays and polymeric‐based delivery systems.

The hydrophobic characteristic of the antibacterial compound influence on which nanostructured clay would be chosen for conjugation without any surface treatment. Antimicrobial agents such as CPC, farnesol, and chlorhexidine were effective in inhibiting cariogenic microorganisms and reducing biofilms. They demonstrated efficacy in release and antibacterial action without disrupting the integrity of the direct restorative dental materials into which they were incorporated. Overall, the incorporation of the functional compounds involved dissolving the nanocarrier powder and then incorporating the agent into this solution for subsequent drying.

This scoping review is distinguished by its innovative approach in mapping and analyzing the use of nanostructured clays in direct restorative dental materials for controlled functional compounds release. While it provides valuable insights into the properties of nanostructured clays and their incorporation into direct restorative dental materials, which are extensively discussed in the literature, it is essential to recognize the limitations due to the scarcity of studies considering them as carriers of functional compounds in these dental materials. Over the past 5 years, only a few studies (*n* = 5) have explored the use of nanostructured clays as carriers for these functional compounds to formulate antibacterial direct restorative dental materials.

Developing materials with nanocarriers has been a crucial tool in addressing the issue of caries adjacent to tooth restorations, highlighting the need for more in vitro studies to further advance anticaries direct restorative dental materials. Detailed methodologies for material manipulation are crucial for this development. The poor details reported in the primary studies raised the need that future research in this area to provide comprehensive descriptions of proportions, materials, and analyses to enable replication of the studies. Establishing a solid foundation of evidence on the mechanical properties and controlled release of antimicrobial agents is essential to support the development of clinical studies.

Although this scoping review did not aim to make clinical recommendations but rather to synthesize the existing literature of a theme, it is important to note that the overall risk of bias in the included studies was moderate. This finding is primarily attributed to methodological shortcomings observed in several of the in vitro studies analyzed. Thus, it becomes even more essential to advocate for the standardization of material characterization protocols in future investigations. Universal testing approaches are critical to ensure the scientific reliability and clinical translational potential of the materials being developed. The development of specific guidelines could solve the gap.

Future studies are encouraged to extend the observation period of controlled release assays to provide stronger support for the development of subsequent clinical investigations. In addition to the in vitro studies already proposed, clinical trials are essential to determine whether the incorporation of nanostructured complexes with antibacterial function can effectively prevent or reduce the occurrence of caries lesions adjacent to the restorative material, an outcome that represents the true purpose of designing antibacterial restorative systems. However, the clinical translation of such materials still depends on further in vitro and in vivo studies to refine their properties and ensure their safety and effectiveness. Moreover, conducting clinical trials presents several challenges, including the need for long‐term follow‐up, potential patient dropout, and the complexity of developing and standardizing novel restorative materials, factors that require careful planning and robust study designs.

This scoping review contributes to a broader understanding of how these materials can be applied to enhance the antibacterial effect of direct restorative dental materials, exploring the advancements in the use of nanostructured clays.

## 5 Conclusion

MMT and HNTs were the main nanostructured clays used for controlled release of functional compounds in direct restorative dental materials. Although MMT is the most commonly used in studies, the literature suggests that HNTs possess properties that facilitate the incorporation of both hydrophilic and hydrophobic compounds without the need for prior treatment, due to their dual layers with different charges. These nanostructured clays, combined with cetylpyridinium chloride, chlorhexidine, and farnesol, demonstrated efficient aggregation. The functional compound‐nanostructured clay systems showed no material degradation, retained good mechanical properties, and were effective against caries‐related oral bacteria without cytotoxicity. Functional compounds were incorporated at concentrations up to 10 wt%, maintaining material integrity and effectiveness, indicating potential for future research. Despite the limited number of studies published in recent years, this review aims to encourage further research by highlighting existing gaps, particularly the need for more in vitro investigations and the promotion of clinical studies using the developed materials. Such efforts are essential to better understand the antibacterial activity of these materials and their potential to prevent or reduce the occurrence of caries lesions adjacent to restorations.

## Author Contributions

**Bárbara Faria de Sa Barbosa:** data curation, investigation, methodology, writing – original draft. **José Leôncio Ferreira Neto:** data curation, investigation, methodology, writing – original draft. **Francisca Jennifer Duarte de Oliveira:** data curation, methodology, writing – original draft. **Juliana Sales Osterno Leitão:** writing – original draft. **Moan Jéfter Fernandes Costa:** supervision, writing – review and editing. **Pedro Henrique Sette‐de‐Souza:** supervision, writing – review and editing. **Pierre Basílio Almeida Fechine:** supervision, writing – review and editing. **Victor Pinheiro Feitosa:** supervision, writing – review and editing. **Boniek Castillo Dutra Borges:** conceptualization, supervision, writing – review and editing.

## Conflicts of Interest

The authors declare no conflicts of interest.

## Data Availability

The authors have nothing to report.
